# Recurrent Stevens–Johnson syndrome/toxic epidermal necrolysis overlap during sequential anti-HER2 therapy in metastatic breast cancer

**DOI:** 10.1016/j.jdcr.2026.08.022

**Published:** 2026-08-22

**Authors:** Josué Vázquez Arizmendi, Guadalupe Estrada Chávez, Gabriel Ernesto Díaz Juárez, Vianey Guadalupe Saldaña Herrera, Rowena López Chávez, Azucena Ocampo Bárcenas

**Affiliations:** a Instituto Estatal de Cancerología “Dr. Arturo Beltrán Ortega”, Acapulco, Guerrero, Mexico; b Departamento de Atención Primaria en Salud, Centro de Investigación de Enfermedades Tropicales (CIET), Universidad Autónoma de Guerrero, Acapulco, Guerrero, Mexico

**Keywords:** antibody–drug conjugates, HER2-positive breast cancer, Stevens–Johnson syndrome, toxic epidermal necrolysis, trastuzumab emtansine

## Introduction

Stevens–Johnson syndrome (SJS) and toxic epidermal necrolysis (TEN) are severe immune-mediated mucocutaneous reactions characterized by extensive keratinocyte apoptosis, epidermal detachment, and mucosal involvement, most commonly triggered by medications.^1^ Although uncommon in oncology, they are associated with significant morbidity and mortality.

In breast cancer, SJS/TEN has been described with cytotoxic chemotherapy, endocrine therapies, targeted agents, and, less frequently, radiotherapy.^2^ Increasing use of targeted therapies and antibody–drug conjugates (ADCs) has led to more reports of severe cutaneous adverse reactions, likely reflecting immune modulation and enhanced antigen presentation.^2^^,^^3^

We report a case of metastatic HER2-positive breast cancer complicated by recurrent severe mucocutaneous reactions, including SJS associated with trastuzumab emtansine (T-DM1) followed by a more severe reaction after subsequent systemic therapy.

## Case presentation

A 46-year-old woman with metastatic hormone receptor–positive/HER2-positive breast cancer (ER 40%, PR 15%, HER2 amplified) presented with a progressive mucocutaneous eruption following systemic therapy. At diagnosis, she had pleural and osteoblastic bone metastases.

She received multiple lines of chemotherapy, HER2-directed, endocrine, and trastuzumab emtansine (T-DM1) therapy (Fig 1). Following progression of central nervous system metastases, T-DM1 was reintroduced at a dose of 260 mg intravenously every 3 weeks together with zoledronic acid. After the third treatment cycle, she developed pruritic erythematous lesions on the lower extremities that rapidly progressed to involve the trunk, face, and upper extremities. The eruption was accompanied by fever and oral and ocular mucosal involvement, requiring hospitalization.

**Fig 1:**
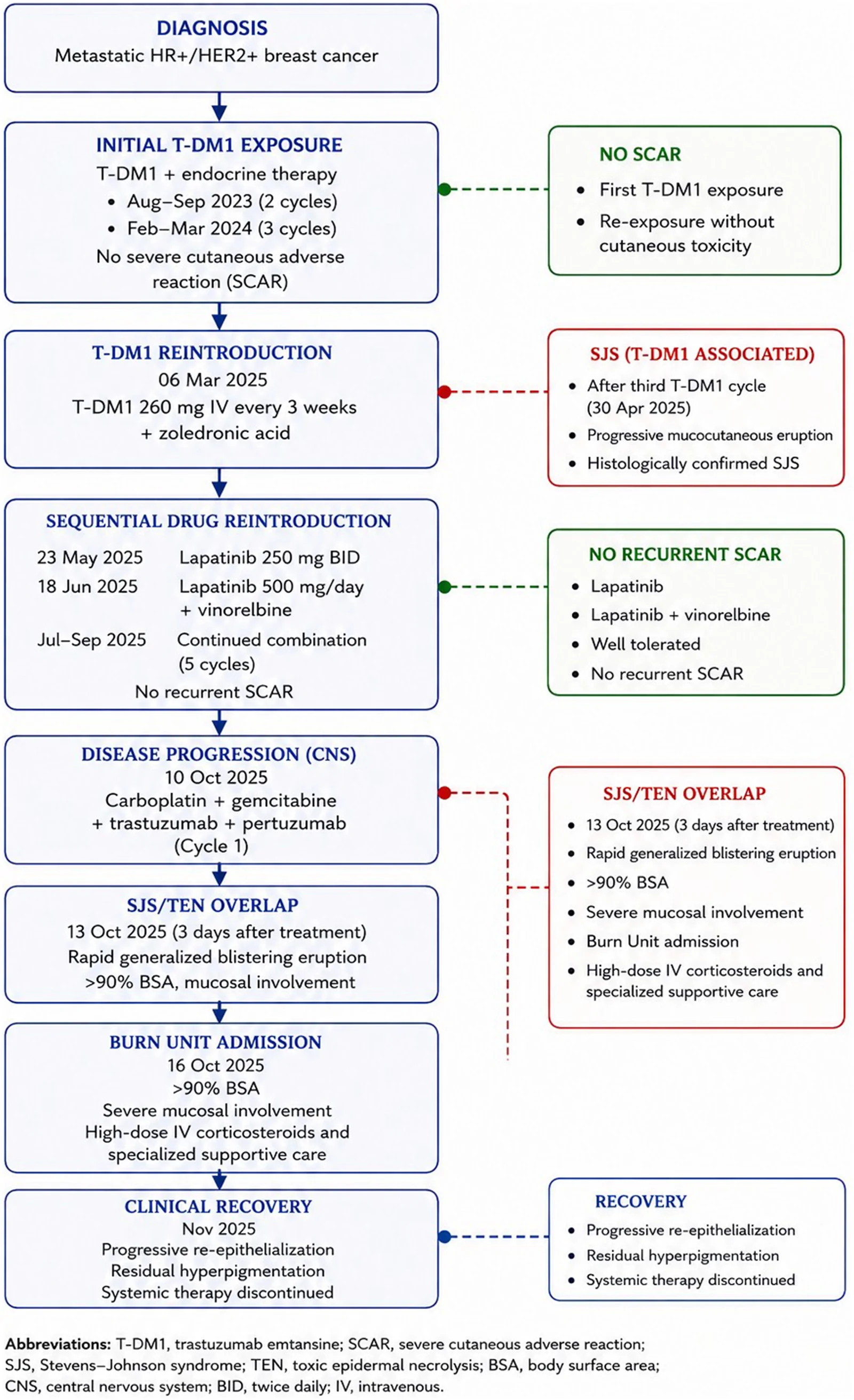
Chronological timeline of systemic therapies, severe cutaneous adverse reactions, and major clinical outcomes.

Physical examination revealed erythematous macules and papules, erosive cheilitis, oral mucosal desquamation, and bilateral conjunctival hyperemia (Fig 2, *A*-*D*). Skin biopsy demonstrated subepidermal blister formation, full-thickness epidermal necrosis, numerous apoptotic keratinocytes, basal vacuolar degeneration, and a predominantly superficial lymphocytic inflammatory infiltrate, consistent with Stevens–Johnson syndrome (Fig 3, *A*-*D*). No eosinophils, epidermal dysmaturation, or ring mitoses were identified. Direct immunofluorescence was not performed. She received systemic corticosteroids and supportive care, with gradual clinical improvement. After recovery, dermatology recommended sequential drug reintroduction. Lapatinib monotherapy was initiated and subsequently escalated to lapatinib plus vinorelbine, both of which were well tolerated without recurrence of cutaneous toxicity.

**Fig 2:**
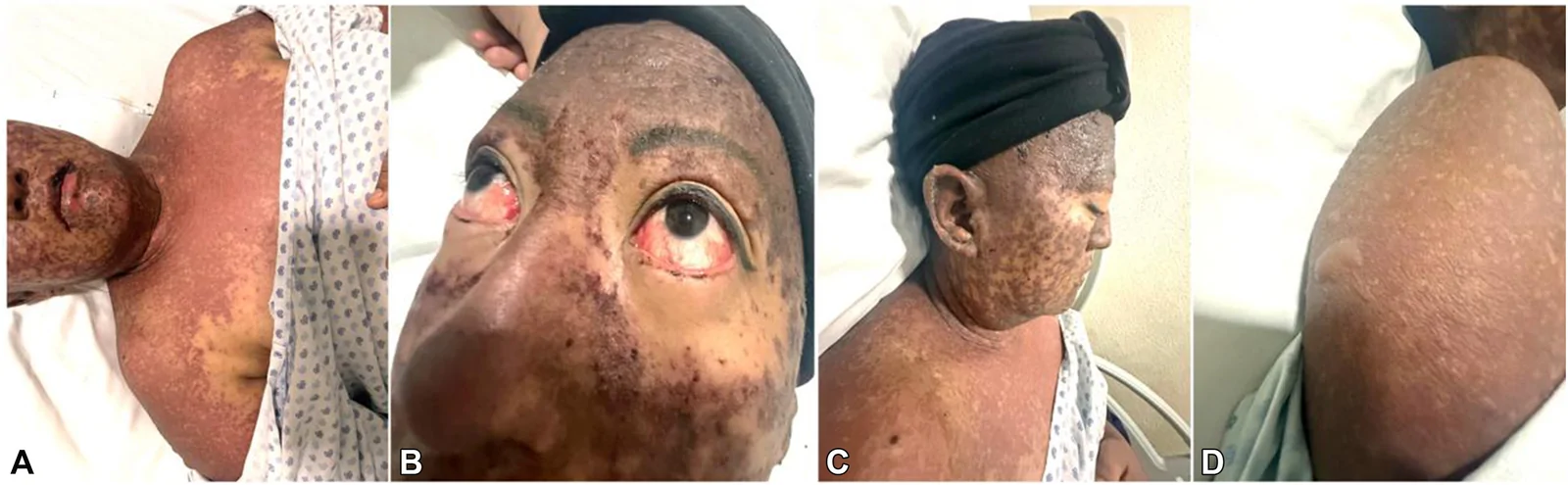
Clinical manifestations during the patient's recurrent severe cutaneous adverse reactions. **A** and **B,** Early mucocutaneous involvement with widespread violaceous macules and patches on the trunk and bilateral conjunctival injection. **C,** Progressive facial and cervical involvement with hyperpigmented lesions. **D,** Advanced stage with epidermal detachment and desquamation.

**Fig 3:**
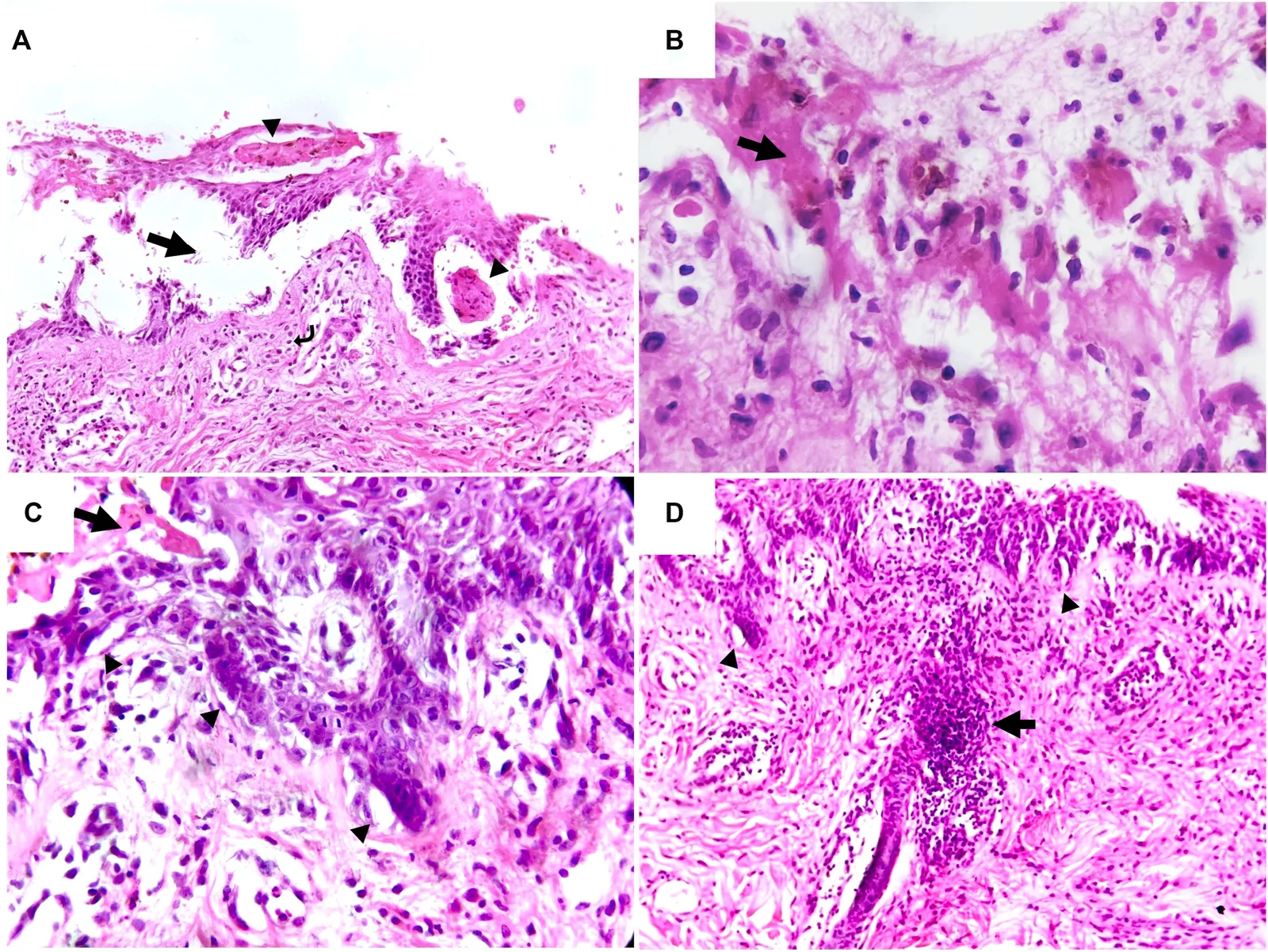
Hematoxylin and eosin (H&E) staining of the skin biopsy demonstrating findings consistent with Stevens–Johnson syndrome. **A,** Low-power view (10×) showing subepidermal blister formation (*arrow*) with extensive epidermal necrosis (*arrowheads*). **B,** High-power view (40×) showing apoptotic keratinocytes (*arrow*). **C,** High-power view (40×) demonstrating basal vacuolar degeneration (*arrowheads*) and apoptotic keratinocytes (*arrow*). **D,** Low-power view (20×) showing a superficial dermal lymphocytic inflammatory infiltrate (*arrow*) beneath the necrotic epidermis (*arrowheads*).

After further progression of central nervous system metastases, treatment was changed to carboplatin, gemcitabine, trastuzumab, and pertuzumab. Three days after the first cycle, she developed a rapidly progressive blistering eruption involving >90% of the body surface area, with extensive epidermal detachment and severe oral, ocular, and genital mucosal involvement. At presentation, she fulfilled 4 SCORTEN criteria, including age >40 years, underlying malignancy, heart rate of 140 beats/min, and >10% body surface area involvement, indicating severe disease. Laboratory evaluation demonstrated anemia (hemoglobin, 9.1 g/dL), mild hypoalbuminemia (3.2 g/dL), and mildly elevated liver enzymes (AST, 64 U/L; ALT, 54 U/L). Serum bicarbonate was unavailable, precluding complete SCORTEN calculation. Clinical findings were consistent with toxic epidermal necrolysis (TEN), with extensive epidermal detachment involving >90% of the body surface area. She was admitted to a burn unit and managed with high-dose systemic corticosteroids and specialized supportive care, with progressive re-epithelialization and clinical improvement (Fig 2).

## Discussion

In HER2-positive breast cancer, SJS/TEN has been reported with trastuzumab, trastuzumab emtansine (T-DM1), lapatinib, and, less frequently, cytotoxic chemotherapy^2^^,^^4^^,^^5^ Increasing use of targeted therapies and antibody–drug conjugates (ADCs) may contribute to these severe cutaneous adverse reactions through immune activation and enhanced antigen presentation^2^^,^^3^ This case documents recurrent severe cutaneous adverse reactions during sequential systemic therapy. The first episode followed T-DM1 reintroduction and was supported by compatible clinicopathologic findings, whereas the second occurred after carboplatin, gemcitabine, trastuzumab, and pertuzumab, progressing to epidermal detachment involving >90% body surface area.

The close temporal relationship between T-DM1 reintroduction and the first episode, compatible clinicopathologic findings, clinical improvement after drug withdrawal, tolerance to subsequent lapatinib, and previous reports strongly support T-DM1 as the most likely culprit. Antibody–drug conjugates such as T-DM1 may promote immune activation through immunogenic cell death, neoantigen exposure, and damage-associated molecular patterns, consistent with a type IVc hypersensitivity reaction mediated by cytotoxic T lymphocytes through granulysin, perforin, and Fas–Fas ligand signaling.^1^^,^^3^

Notably, sequential lapatinib monotherapy followed by lapatinib plus vinorelbine was well tolerated without recurrence of severe cutaneous toxicity. Although shared immune recognition among trastuzumab-containing therapies cannot be excluded, this observation supports T-DM1 rather than HER2 blockade itself as the most likely trigger, arguing against a uniform class effect.

Interpretation of the second episode is more challenging because carboplatin, gemcitabine, trastuzumab, and pertuzumab have each been rarely associated with severe cutaneous adverse reactions.^2^^,^^4^^,^^5^ Attribution to a single culprit drug is therefore not possible. While immune priming may have contributed to the increased severity, concurrent exposure to multiple agents precludes establishing immune reactivation as the sole pathogenic mechanism. Persistence of drug-specific cytotoxic T-cell populations has been proposed as a mechanism for enhanced responses following subsequent immunologic stimulation.^1^

The differential diagnosis included toxic erythema of chemotherapy (TEC), erythema multiforme major, generalized bullous fixed drug eruption, and paraneoplastic pemphigus. TEC was considered because ADCs and other anticancer therapies may produce TEC-like eruptions; however, the extensive epidermal necrosis, prominent mucosal involvement, compatible histopathologic findings, and rapid clinical progression favored SJS/TEN over these alternative entities.^6^

Compared with previously reported cases of SJS/TEN associated with individual HER2-targeted therapies^4^^,^^5^ or other targeted agents such as alpelisib,^7^ this report documents recurrent severe cutaneous adverse reactions during sequential oncologic therapies in a patient with metastatic HER2-positive breast cancer. A previous history of SJS/TEN should prompt careful assessment of culprit drugs, close dermatologic monitoring, and multidisciplinary management during subsequent systemic therapy.

In our patient, prompt discontinuation of systemic therapy, high-dose corticosteroids, and specialized supportive care were associated with clinical recovery, consistent with current recommendations.^8^ Nevertheless, multiple concomitant agents precluded identification of a single culprit drug. ALDEN was not performed, and immunologic and pharmacogenetic susceptibility markers were unavailable. Despite these limitations, the chronological sequence, histopathologic confirmation of the initial episode, and recurrence during subsequent therapy provide clinically relevant evidence for similar patients receiving HER2-directed therapies.

## Conflicts of interest

None disclosed.
